# Phylogenetic and Biogeographic Analysis of Sphaerexochine Trilobites

**DOI:** 10.1371/journal.pone.0021304

**Published:** 2011-06-27

**Authors:** Curtis R. Congreve, Bruce S. Lieberman

**Affiliations:** Department of Geology and Biodiversity Institute, University of Kansas, Lawrence, Kansas, United States of America; Field Museum of Natural History, United States of America

## Abstract

**Background:**

Sphaerexochinae is a speciose and widely distributed group of cheirurid trilobites. Their temporal range extends from the earliest Ordovician through the Silurian, and they survived the end Ordovician mass extinction event (the second largest mass extinction in Earth history). Prior to this study, the individual evolutionary relationships within the group had yet to be determined utilizing rigorous phylogenetic methods. Understanding these evolutionary relationships is important for producing a stable classification of the group, and will be useful in elucidating the effects the end Ordovician mass extinction had on the evolutionary and biogeographic history of the group.

**Methodology/Principal Findings:**

Cladistic parsimony analysis of cheirurid trilobites assigned to the subfamily Sphaerexochinae was conducted to evaluate phylogenetic patterns and produce a hypothesis of relationship for the group. This study utilized the program TNT, and the analysis included thirty-one taxa and thirty-nine characters. The results of this analysis were then used in a Lieberman-modified Brooks Parsimony Analysis to analyze biogeographic patterns during the Ordovician-Silurian.

**Conclusions/Significance:**

The genus *Sphaerexochus* was found to be monophyletic, consisting of two smaller clades (one composed entirely of Ordovician species and another composed of Silurian and Ordovician species). By contrast, the genus *Kawina* was found to be paraphyletic. It is a basal grade that also contains taxa formerly assigned to *Cydonocephalus*. Phylogenetic patterns suggest Sphaerexochinae is a relatively distinctive trilobite clade because it appears to have been largely unaffected by the end Ordovician mass extinction. Finally, the biogeographic analysis yields two major conclusions about *Sphaerexochus* biogeography: Bohemia and Avalonia were close enough during the Silurian to exchange taxa; and during the Ordovician there was dispersal between Eastern Laurentia and the Yangtze block (South China) and between Eastern Laurentia and Avalonia.

## Introduction

The Cheiruridae are a diverse family of phacopine trilobites with a long geologic history spanning the latest Cambrian to the Middle Devonian. Although the group is believed to be monophyletic, the individual species level relationships are largely unknown due to a paucity of phylogenetic studies within the group [1], [2]. Lane [3] provided the most recent taxonomic revision of the entire family, and recognized seven subfamilies within the Cheiruridae. One subfamily is the diverse, Ordovician-Silurian Sphaerexochinae; it is diagnosed by its possession of a wide axis, three pairs of glabellar furrows with S1 being longer and more incised than S2 or S3, eyes positioned close to the axial furrows, triangular free cheek, wide and short rostral plate, thoracic and pygidial doublure extending to the axial furrow, and a hypostome with small anterior wings and a gently inflated middle body and a shallow notch on the posterior border [4]. There are four genera that are readily referable to this subfamily, and a phylogenetic analysis of these will be the focus of this study. The first genus is the eponymous *Sphaerexochus*; the monophyly of *Sphaerexochus* is supported by several apomorphies including a highly inflated glabella that is subcircular in outline, S1 deeply incised and curving sharply towards L0, S2 and S3 faintly incised, free cheeks small and vertical, and a hypostome that is trapezoidal in outline [3], although monophyly had not been previously tested using a phylogenetic approach. It has been proposed that the genus can be further divided into four subgenera (two of which are monotypic): *S. (Sphaerexochus)*, *S. (Korolevium)*, *S. (Parvixochus)*, and *S. (Onukia)* [5], [6]. Three other sphaerexochine genera are *Kawina*, *Cydonocephalus*, and *Forteyops*. *Kawina* had previously been treated as closely related to *Sphaerexochus* on the basis of reduced triangular free cheeks, wide axis of the exoskeleton, eyes situated close to the axial furrow, rostral plate wide (transverse) and short, S1 furrows deeper and longer than S2 and S3, a pygidial and thoracic doublure extending to the axial furrow, and a pygidium with two to three axial rings, a semi-circular outline, a pronounced terminal axial piece, and three pleural spines [4]. Adrain and Fortey [7] reclassified *Cydonocephalus* as a junior synonym of *Kawina*, see also Jell and Adrain [8] **.** The type species of the monotypic *Forteyops*, *Forteyops sexapugia*, was originally grouped within *Kawina* [9] until Pribyl *et al.* [6] moved it into its own genus. The monophyly and relationships of these genera have not been previously tested using a phylogenetic approach. One taxon formerly assigned to the sphaerexochines by Lane [3], *Hyrokybe*, has since been shown to be more closely related to a different cheirurid subfamily: the Acanthoparyphinae (see [1]), and therefore will not be considered herein. The affinities of *Nieszkowskia* also likely lie with this subfamily as well. Other taxa that have been previously assigned to the Sphaerexochinae, such as *Xystocrania* and *Pompeckia*, are either known from limited material or have dubious affinities, and therefore will not be considered herein. Here we present a phylogenetic analysis of the Sphaerexochinae as part of a larger systematic revision of the Cheiruridae, and use the resulting phylogeny to consider biogeographic patterns spanning the Ordovician-Silurian and discern patterns of survival during the end Ordovician mass extinction.

The end Ordovician mass extinction event is considered to be the second largest mass extinction in the history of life and is classically interpreted as being caused by a brief, unstable icehouse during otherwise greenhouse conditions [10]–[12]. The event is particularly important for trilobites, as its selectivity profoundly affected the evolution of the group. Previous research suggests that trilobites with a planktonic larval stage are more strongly affected by the extinction event than trilobites with benthic larvae [13]. Furthermore, trilobite groups with a presumed pelagic adult stage completely go extinct at the Ordovician-Silurian boundary. To put this research into a broader context, the sphaerexochines are a group that survives the event and they have been interpreted as having benthic larvae [14].

## Materials and Methods

### Phylogenetic Analysis

**Figure 1:**
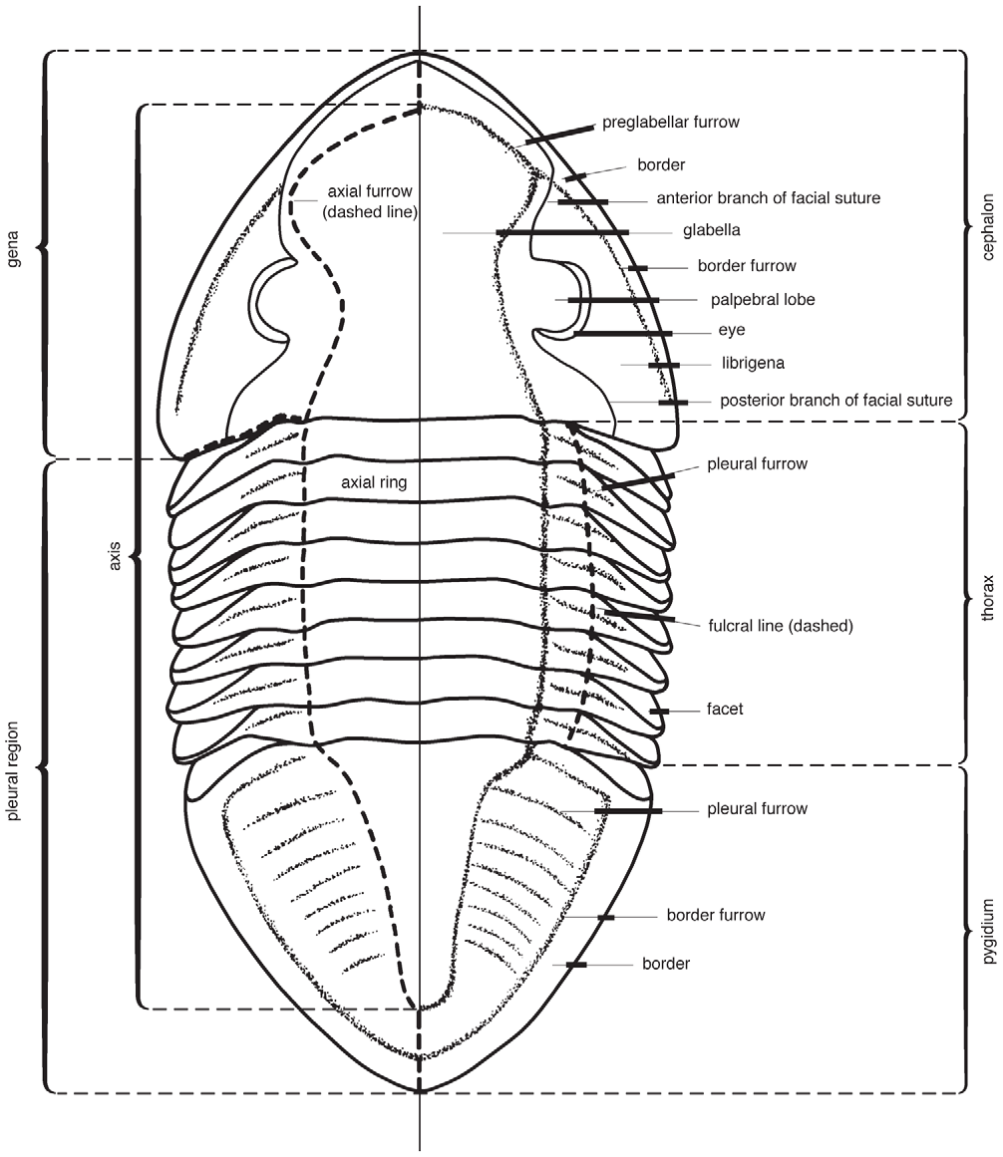
Line drawing of the Ordovician trilobite *Isotelus* with basic anatomical parts labeled. From Treatise on Invertebrate Paleontology, courtesy of ©1997, The Geological Society of America and The University of Kansas.

**Figure 2:**
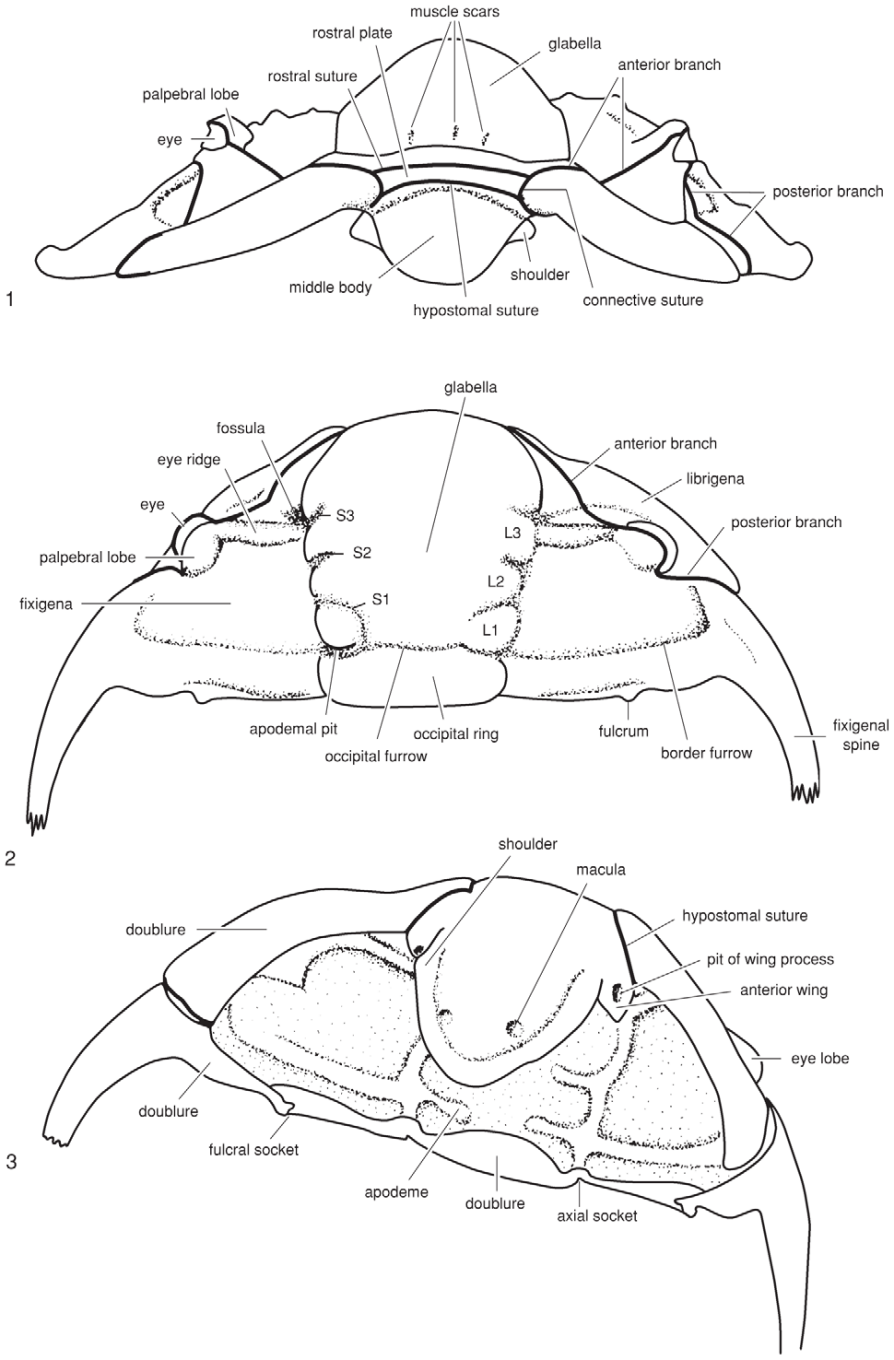
Line drawing of the cephlon of Ordovician cheirurid trilobite *Ceraurus monteyensis* with anatomical terminology labeled. From Treatise on Invertebrate Paleontology, courtesy of ©1997, The Geological Society of America and The University of Kansas.

Morphological terminology follows Whittington et al. [15] (see Fig. 1 and Fig. 2 for line drawings of trilobites with the relevant parts labeled). Material was examined from the Yale University Peabody Museum of Natural History (YPM), the Museum of Comparative Zoology, Harvard University (MCZ), the University of Kansas Museum of Invertebrate Paleontology (KUMIP), the Naturhistoriska Riksmuseet, Stockholm, Sweden (AR), the Paleontological Museum of the University of Oslo, Norway (PMO), the VSEGEI in Saint Petersburg, Russia, and the Swedish Geological Survey, Uppsala (SGU).

#### Taxa Analyzed

A total of thirty-one taxa were included in this analysis. *Forteyops* s*exapugia* was chosen as the outgroup as it appears to represent a basal sphaerexochine distinguished by its very early stratigraphic appearance, and it can be distinguished from all other ingroup sphaerexochines by its possession of dagger-shaped pygidial pleurae (a character common in other cheirurid trilobites but not found in other sphaerexochine species). All well-preserved members of the Sphaerexochinae for which material was available were considered in the phylogenetic analysis. Meriting special mention here is *Sphaerexochus britannicus,* which was included as a distinct species despite Thomas's [16] claim that the species is a synonym of *S. mirus*. Although Thomas [16] argued that the differences in the proportions of the pygidia between the two forms arose solely because the pygidia represented different developmental stages, Ramsköld [17] demonstrated that these differences could not be attributed to ontogenetic changes. Another noteworthy species is *S. scabridus*. Ramsköld [17] argued that this species might be dimorphic as he identified a few long-spined pygidia. However, he noted that these long-spined specimens were rare and that there was insufficient material to conclude if these specimens were in fact dimorphs of *S. scabridus* or if instead they belonged to a different species. Following his cautions, in our analysis we coded only the short-spined specimens of *S. scabridus*, since these are most similar to the neotype established by Ramsköld [17]. Finally, special mention should be given to the potentially dimorphic species, *S. dimorphus*. This taxon was analyzed twice in our study, the first time coding the species as a dimorphic taxon and the second time with each morph coding as a separate species. This procedure investigated whether or not there was sufficient phylogenetic evidence to split *S. dimorphus* into two separate species. Because the character codings for each of the two morphs were distinct from the codings of all other taxa considered in the analysis, these morphs could not be synonymized with any other species. The net relationships suggested by both of these two analyses are identical; however, the analysis that split the two morphs into separate species had slightly worse resolution, with both morphs grouped together in a large polytomy. Coupling this result with the fact that the two morphs only differ in three of the characters used for phylogenetic analysis, we chose to treat *S. dimorphus* as a single dimorphic taxon for the purposes of this paper.

#### Specific Taxa Analyzed

**Figure 3:**
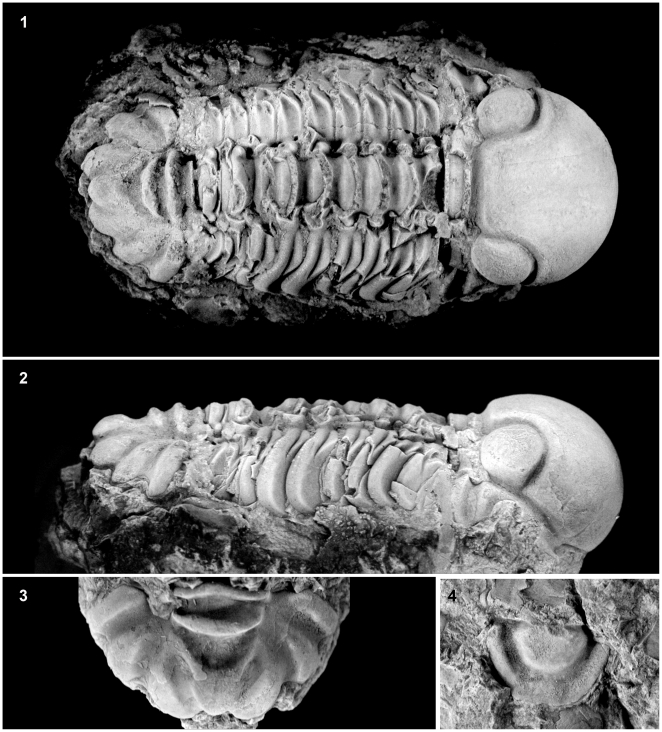
*1*) Complete specimen of *Sphaerexochus mirus* (MCZ 196479) in dorsal view, 3x. *2*) Complete specimen of *Sphaerexochus mirus* (MCZ 196479) in lateral view, 2.25x. *3*) Pygidium of *Sphaerexochus mirus* (MCZ 196498), 3.2x. *4*) Hypostome of *Sphaerexochus mirus* (MCZ196484), 3.25x.

(Relevant material examined is listed where appropriate. In instances where museum material was not examined, species were coded using photographs from scientific publications.) *Forteyops* s*exapugia* (YPM 18289, 18291, 18293); *“K.” arnoldi*; *“K.” divergens*; *K. vulcanus* (YPM 170174, 227101, 227109–227112); *“K.” griphus*; *“K.” torulus*; *“K.” prolificus*; *“K.”mercurius; “K.” scrobiculus; “K.” prominulus; Sphaerexochus latifrons* (AR 30060, 30063, 30065, 30067, 51316–51318, 51320–51322, 51324; YPM 183971–183973); *S. molongloensis*; *S. scarbridus* (AR 29991, 30016, 30042, 30068, 30074, 30075, 30078, 30144, 30187, 30193, 30194, 51305, 51307–51309, 51311–51313, 51315, 51338–51343, 53232–53236; SGU 1401, 1402); *S. atacius*; *S. eurys*; *S. calvus* (SGU 4133–4135; AR 11256–11263, 11375, 11376, 49250); *S. laciniatus* (AR 29831, 29857, 29858, 29860, 29862, 29866, 29882, 30059, 30072, 30112, 51325); *S. johnstoni*; *S. mirus* (AR 39276, 39477–39482, 39484–39486, 39553 a, b; MCZ 1325, 1328, 196479, 196484, 196498; YPM 6573, 183982–183984, 183998–194000) (Fig. 3); *S. britannicus*; *S. pulcher*; *S. parvus*; *S. brandlyi*; *S. romingeri* (KUMIP 105187–105190; MCZ 195135, 195139, 195144, 195146, 195190, 195195, 195535, 195541, 195546, 195548, 195555, 195565; YPM 183978–183981, 184003); *S. fibrisulcatus*; *S. hapsidotus*; *S. dimorphus*; *S. glaber*; *S. hiratai*; and *S. arenosus.*

#### Characters

The characters used in phylogenetic analysis are listed below in approximate order from anterior to posterior position on the organism. A complete character matrix is given in Table S1. Characters emphasize the adult, holaspid stage as there are only a limited number of taxa for which early ontogenetic stages are available.

Cephalon

1. S1; 0: contacts S0, 1: does not contact S0.
2. Space between the proximal edges of both L1 lobes measured transversely (dorsal view); 0: wide (distance between the proximal edges of L1/posterior glabellar margin transverse width = 0.5), 1: narrow (distance between the proximal edges of L1/posterior glabellar margin transverse width = 0.33).
3. Point of maximum glabellar convexity (lateral view); 0: medial, 1: anterior.
4. S2 and S3; 0: strongly incised, 1: weakly incised, 2: indistinct or absent.
5. Genal spines; 0: present; 1: absent or reduced to small thorn-like projections.
6. Angle formed by the intersection of the anterior and lateral glabellar margins, in anterior view; 0: relatively broad (115–120 degrees), 1: relatively narrow (105–110 degrees).
7. Tubercle on center of L0; 0: present, 1: absent.
8. Shape of S1 close to the lateral glabellar margins; 0: S-shaped, 1: straight.
9. Border of librigena; 0: wide (ratio of exsagittal width of librigena to border width is 0.4–0.5); 1: narrow (ratio of exsagittal width of librigena to border width is 0.2–0.33).
10. L0; 0: wide (maximum glabellar width (tr.)/L0 (tr.) is 1.2–1.4), 1: narrow (maximum glabellar width (tr.)/L0 (tr.) is 1.6–1.8).
11. S1; 0: strongly incised, 1: weakly incised to indistinct.
12. Anterior glabellar margin (in anterior view); 0: roughly straight, 1: strongly convex.
13. Glabella between S0 and S1 (in lateral view); 0: curves uniformly with the rest of the glabella, 1: inflates dramatically.
14. S1 orientation; 0: runs roughly transverse, 1: curves posteriorly.
15. Shape of medial part of S0; 0: straight, 1: concave anteriorly.
16. Lateral margins of the glabella immediately anterior of S1 (in dorsal view); 0: roughly parallel, 1: strongly converging, 2: strongly diverging.
17. Border furrow on librigena; 0: pencil thin (ratio of exsagittal width of librigena to border furrow width is 0.1), 1: narrow (ratio of exsagittal width of librigena to border furrow width is 0.15–0.22), 2: wide (ratio of exsagittal width of librigena to border furrow width is 0.27–0.33).

Hypostome

1. Middle body furrow; 0: does not intersect or only faintly contacts outer border furrow, 1: prominently intersects outer border furrow.
2. Middle body furrow of hypostome; 0: prominently intersects entire middle body, 1: restricted to the lateral edges of the middle body.
3. Posterior margin; 0: possesses a strongly concave pocket, 1: is straight or with concave pocket strongly reduced to absent.

Pygidium (Note, all measurements of the terminal axial piece use the notch on the lateral edges of the terminal axial piece as the anteriormost point of the axial piece if the segment has been fused to the axial ring.)

1. Pleural spines; 0: terminate close to each other, forming a pygidial shield, 1: separate from each other distally.
2. Inter-pleural furrows; 0: wide, 1: narrow (pencil thin).
3. Anteriormost set of pleural spines; 0: has proximal “kink” associated with a 60–80 degree angle change and a long crescent shaped notch on the anterior side of the spine, 1: gradually curves proximally, with the notch absent or reduced.
4. Distal pleural tips; 0: flat, 1: rounded, 2: subtriangular.
5. Width (tr.) of terminal axial piece; 0: narrow (tr.) (transverse width of the anteriormost part of the axial piece ∼ three quarters of its length (sag.)), 1: wide (tr.) (transverse width of the anteriormost part of the axial piece ∼ two times its length (sag.)), 2: average (tr.) (transverse width of the anteriormost part of the axial piece ∼ its length (sag.)).
6. Pygidial convexity (posterior view); 0: vaulted, 1: nearly flat.
7. Pygidial dimensions; 0: wide and short (pygidial width (tr.) divided by length (sag.) is roughly 2.1–2.2), 1: long and narrow (pygidial width (tr.) divided by length (sag.) is roughly 1.6–1.8), 2: very long (pygidial width (tr.) divided by length (sag.) is roughly 1–1.3).
8. First axial ring; 0: wide (width (tr.) of axial ring divided by width (tr.) of pleural field ∼1.5–1.7), 1: narrow (width (tr.) of axial ring divided by width (tr.) of pleural field ∼1).
9. Posteriormost part of terminal axial piece in dorsal view; 0: rounded, 1: pointed.
10. Maximum convexity of terminal axial piece, in lateral view; 0: anterior, 1: medial, 2: posterior.
11. Interpleural furrows; 0: deep, 1:shallow.
12. Lateral margins of second set of pleural spines at approximate spine midpoint; 0: strongly curved, 1: weakly curved to straight.
13. Terminal axial piece size; 0: small (length (sag.) < length (sag.) of first axial ring), 1: large (length (sag.) >1.5 length (sag.) of first axial ring).
14. Distal tips of pleural spines; 0: hooked (i.e., sharply curved near distal ends), 1: straight.
15. Distal ends of the posteriormost pleural spines; 0: dramatically inflate laterally, 1: remain relatively the same size.
16. Angle the pygidial axial furrow along axial ring 1 and 2 forms with a sagittal line, 0: shallow (∼20°), 1: sharp (>30°).
17. Furrow on proximal end of first pleural spine; 0: visible in dorsal view, 1: not visible in dorsal view.
18. Lateral edges of terminal axial piece; 0: straight sided, 1: strongly curved.
19. Third axial ring; 0: fused completely to terminal axial piece, forming a notch, 1: partially fused (ring partly visible), 2: ring distinct (not fused).

#### Methods

The data were analyzed using TNT v1.1 [18]. A traditional search algorithm using TBR with 10,000 replications, 1 random seed, and 10 trees saved per replication was used to determine the most parsimonious trees for the data matrix. All characters were unweighted and all multistate characters were treated as unordered as there were no obvious criteria for ordering them. To assess tree support, bootstrap, jackknife, and Bremer [19] support values were calculated in TNT. Bootstrap and jackknife tests were analyzed using 10,000 replicates and a traditional search (13 characters, 33 percent of the data, were removed during the jackknife test). The matrix data were compiled into Nexus files using Mesquite v.2.01 [20] and trees were generated using FigTree v.1.1.2 [21].

### Biogeographic Analysis

**Figure 4:**
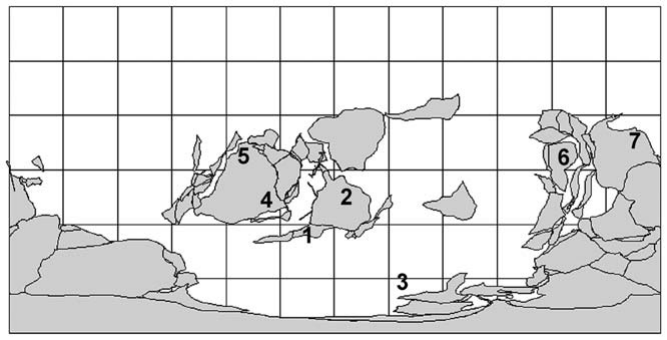
Paleogeographic reconstruction for the Late Ordovician (Caradoc) generated using ArcView 9.2 and PaleoGIS [**50**]. The biogeographic areas used in this analysis are shown where *1* = Avalonia, *2* = Baltica, *3* = Bohemia, *4* = Eastern Laurentia, *5* = Northwestern Laurentia, *6* = Yangtze block, and *7* = Australia.

**Figure 5:**
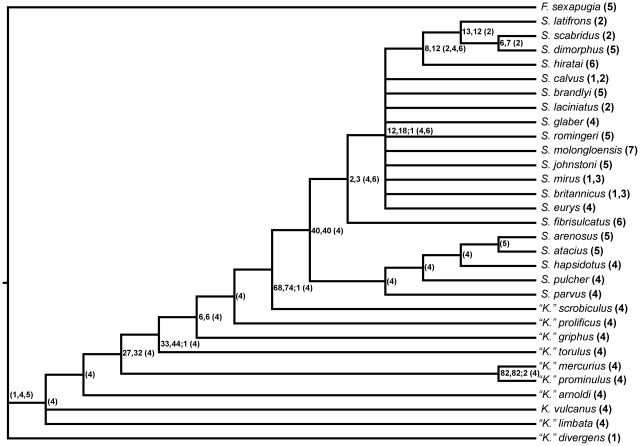
Results from parsimony analysis showing strict consensus of 29 most parsimonious trees of length 116 steps. Tree graphics generated using FigTree v.1.1.2 [21] with genera labeled and paraphyletic genus identified using quotations following Wiley [39]. The values written in plain text are bootstrap, jackknife, and Bremer Support values respectively; the values that are bracketed, i.e., (*1, 2*, …), are the areas used in the biogeographic analysis, coded as follows: *1* = Avalonia; *2* = Baltica; *3* = Bohemia; *4* = Eastern Laurentia; *5* = Northwestern Laurentia; *6* = Yangtze; and *7* = Australia.

The results from phylogenetic analysis were used in biogeographic analysis by applying Lieberman-modified Brooks Parsimony Analysis (LBPA) [22]. This method is described in detail in Lieberman and Eldredge [23], and Lieberman [24], [25]; the method has been used to investigate biogeographic patterns in a variety of fossil taxa, (e.g. [23], [24], [26]–[28]). An area cladogram was created by replacing the names of the terminal taxa on the consensus most parsimonious tree with the geographic areas where these taxa were found. The areas used in the analysis are: Avalonia (present day Great Britain and Ireland); Eastern (E.) and Northwestern (N.W.) Laurentia; Bohemia (Central Europe); Yangtze block (South China and Japan); Australia; and Baltica (present day Norway, Sweden, eastern Russia, and Finland) (Fig. 4). These areas represented distinct geological regions and also contained large numbers of endemic taxa during the Ordovician and Silurian; in effect these definitions follow the area designations of Scotese and McKerrow [29], Fortey and Cocks [30], Harper [31], Torsvik et al. [32], [33], and Zhou and Zhen [34]. One of the species used in the analysis, *Sphaerexochus eurys*, is found in Scotland (in the Midland Valley Terrane- Girvan). For the purposes of this analysis, Scotland was treated as part of Eastern Laurentia based on paleomagnetic and faunal studies that suggest the Midland Valley Terrane stayed peripheral to Laurentia throughout the Ordovician [33], [35]. Next, the geographic locations for the ancestral nodes of the area cladogram were optimized using a modified version of the Fitch [36] parsimony algorithm (Fig. 5). The area cladogram was then used to generate two data matrices, one to code for congruent patterns of geodispersal (Table S2) and the other to code for congruent patterns of vicariance (Table S3). The former provides information about the relative time that barriers formed, isolating regions and their respective biotas; the latter provides information about the relative time that barriers fell, allowing biotas to congruently expand their range [23]–[25]. Each matrix was then analyzed using the exhaustive search function of PAUP* 4.0 [37] as well as the implicit enumeration function in TNT v1.1 [18]. PAUP* was used in addition to TNT because the exhaustive search function in PAUP* calculates the g_1_ statistic, which can be used to gauge whether the results were significantly different from randomly generated data. Both programs yielded the same trees. The method results in the generation of two trees, one tree showing congruent patterns of range expansion (the geodispersal analysis) and the other tree showing congruent patterns of range contraction (the vicariance analysis).

## Results

### Phylogenetic Analysis

**Figure 6:**
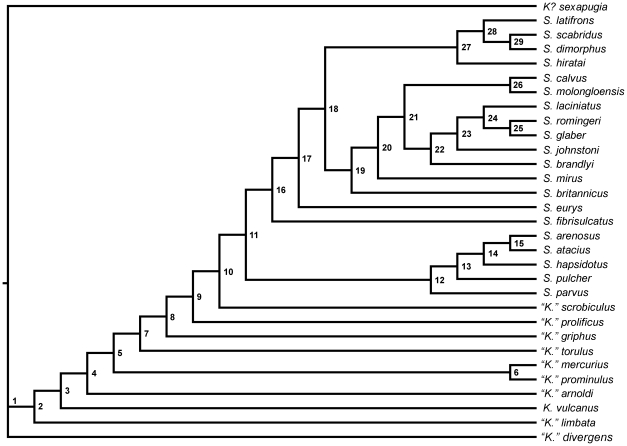
One of the 29 most parsimonious trees with characters mapped on the tree; parentheses denote unambiguous optimizations and curly brackets denote ambiguity. *Node 1*∶1 {0,1}; 5 {0,1}; 28 (1). *Node 2*∶1 (1); 5 (1); 16 (1); 21 {0,1}; 27 (2); 28 (0). *Node 3*∶9 {0,1}; 21 (0). *Node 4*∶15 (1); 19 (1); 33 (1); 34 (0). *Node 5*∶3 (1); 9 (1); 24 {0,1,2}; 27 {1,2}; 39 {1,2}. *Node 6*∶13 (1); 14 (1). *Node 7*∶6 (0); 17 (1). *Node 8*∶7 (0). *Node 9*∶16 (0). *Node 10*∶4 (1); 5 {0,1}; 12 (1). *Node 11*∶2 (0); 5 (0); 8 (1); 27 (1) 39 (1). *Node 12*∶9 {0,1}; 20 (0); 31 (0). *Node 13*∶21 (1); 24 (1); 27 (0); 35 {0,1}. *Node 14*∶22 {0,1}; 23 {0,1}; 30 {0,1}; 36 (1); 37 (0). *Node 15*∶9 (1); 12 (0); 16 (2); 33 (0). *Node 16*∶4 (2); 24 (1); 26 {0,1}. *Node 17*∶1 (0); 10 (1); 26 (0); 29 (0). *Node 18*∶38 (1); 39 (0). *Node 19*∶32 (0). *Node 20*∶22 (0). *Node 21*∶38 (0). *Node 22*∶30 (0). *Node 23*∶21 {0,1}; 23 (0). *Node 24*∶17 (2); 32 {0,1}. *Node 25*∶25 (0); 31 (0). *Node 26*∶9 (0); 35 (0). *Node 27*∶34 (0). *Node 28*∶31 (0). *Node 29*∶27 (0).

The analysis generated 29 most parsimonious trees of length 115 steps, with CI (excluding uninformative characters) and RI values of 0.405 and 0.715 respectively. A strict consensus of these trees (Figs. 5, 6) suggests that taxa traditionally assigned to *Kawina* and *Cydonocephalus* form a paraphyletic grade at the base of a monophyletic *Sphaerexochus*. Species relationships within part of *Sphaerexochus* are uncertain; however, there are at least two smaller clades within this monophyletic group, with the more resolved clade consisting entirely of mid Ordovician species and the polytomy consisting of Silurian species and the Ordovician species *S. calvus*, *S. fibriculatus,* and *S. eurys*.

Part of the lack of resolution in Silurian *Sphaerexochus* can be attributed to *S. romingeri*. If this taxon is removed from the analysis, TNT generates a single most parsimonious tree of 112 steps. However, since *S. romingeri* is well preserved and known from ample material, there seems to be no clear grounds for excluding it from the analysis.

### Biogeographic Analysis

#### Results of the analysis

**Figure 7:**
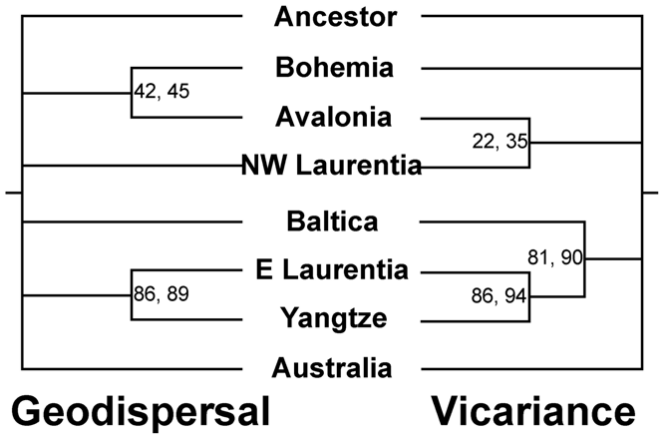
Results from biogeographic analysis: on the left the strict consensus of the three most parsimonious geodispersal trees; and on the right the most parsimonious vicariance tree.

The LBPA yielded three most parsimonious geodispersal trees of length 69 steps (Fig. 7). The strict consensus of these three trees results in two resolved nodes uniting E. Laurentia and the Yangtze Block, and Avalonia and Bohemia. In addition, a single most parsimonious vicariance tree of length 60 steps was also recovered. The tree has three resolved nodes uniting N.W. Laurentia and Avalonia, E. Laurentia and the Yangtze Block, and Baltica with a combined E. Laurentia-Yangtze Block.

We used the test of Hillis [38], the g_1_ statistic, to determine whether the results of our analysis differ from those produced by random data. The g_1_ statistics for the geodispersal and vicariance components of the LBPA were −1.175 and −1.005 respectively, suggesting that our results differ from random data with a significance value of 0.01.

## Discussion

### Phylogenetic Analysis

Our analysis suggests that the genus *Sphaerexochus* as originally defined is monophyletic. By contrast, *Kawina* and *Cydonocephalus* form one large grade at the base of *Sphaerexochus*, and are therefore paraphyletic. We suggest that *Kawina* be redefined as a monotypic genus including its type species *K. vulcanus,* and that all other species originally placed within *Kawina* and *Cydonocephalus* be placed within *“Kawina*”, with the quote marks denoting paraphyly following the convention of Wiley [39]. We are hesitant to lump all of the species originally assigned to *Kawina* and *Cydonocephalus* into *Sphaerexochus* because relationships within *“Kawina”* may be more complex than this analysis suggests. Therefore, we consider it prudent to differentiate them from *Sphaerexochus* until a larger scale phylogeny can piece apart the complete taxonomic relationships of other taxa including *Xystocrania* and *Nieszkowskia*.

The topology suggests that there are at least two smaller clades within the genus *Sphaerexochus*, giving partial support to some of the previously identified subgeneric groupings within the genus (*sensu* [6]). The smaller of the two clades contains *S. arenosus*, *S. atacius*, *S. pulcher*, and *S. parvus*, all of which are species that were originally placed by Pribyl *et al.* [6] into the subgenera *S. (Korolevium)* and *S. (Parvixochus)*. Thus, *S. (Korolevium)* as it was originally defined is a monophyletic group. The monotypic *S. (Parvixochus)*, which contains the species *S. parvus*, maps basally to the *S. (Korolevium)* clade. Since the placement of *S. (Parvixochus)* on the tree does not reduce *S. (Korolevium)* to a paraphyletic group, there is no sufficient evidence to suggest synonymy of these two groups. The second clade consists of species placed within *S. (Sphaerexochus)* and is also monophyletic as originally defined. The phylogenetic placement of the monotypic subgenus *S. (Onukia)* [5] could not be assessed in this analysis because the type species of the group is based on poorly preserved and deformed specimens that could not be analyzed phylogeneticly.

The tree topology suggests that the genus *Sphaerexochus* was relatively unaffected by the mass extinction event at the end Ordovician. While the *S (Korolevium)*-*S. (Parvixochus)* clade consists entirely of Ordovician species, these species go extinct during the Middle Ordovician so the mass extinction event cannot be invoked to explain the clade's disappearance. The *S. (Sphearexochus)* clade contains Silurian and Ordovician species and further supports the hypothesis that this group passed through the mass extinction event essentially unscathed. Even though our analysis of this clade resulted in a large polytomy, no matter how the polytomy is resolved, the implications are generally the same. For instance, if the Ordovician species map out basal to the Silurian species, this implies that the common ancestors of these Ordovician species survived the event and gave rise to the new Silurian forms. If, however, one or more of the Ordovician species maps nested within the Silurian species, then it implies that the diversification within the Silurian clade has its roots in the Middle or Late Ordovician. This is true even if *S. romingeri* is removed from the analysis because then the taxon *S. calvus* (a Late Ordovician species) maps out nested within the Silurian forms; this would actually suggest that the end Ordovician may have been a time of significant diversification for species within *Sphaerexochus*. This type of extinction resistance of select groups of trilobites across the end Ordovician mass extinction is not entirely unique, as it has been observed by other authors, such as Adrain *et al.* [40].

Phylogenetic analysis reveals interesting patterns of character evolution within *Sphaerexochus*. Holloway [41] argued that Silurian species of *Sphaerexochus* showed little difference in their cephala, concluding that the cephalon could not provide diagnostic characters for species identification in Silurian species. Our results mirror this assessment: species grouping within the large Silurian polytomy all code similarly for cephalic characters, varying only in the width of the free cheek border and the width of the free cheek furrow. This fixation in the characters of the cephalon might suggest an evolutionary bottleneck. Potentially, one source of that bottleneck might be preferential extinction at the end Ordovician. However, the phylogeny suggests that the *Sphaerexochus* clade was not especially affected by the mass extinction event; instead, the bottleneck might have occurred in the Middle Ordovician. It is also possible that the fixation of cephalic characters could be due to some sort of evolutionary burden or developmental constraints *sensu* Riedl [42] and Gould [43], but such a possibility at this time remains untestable.

### Systematic Paleontology

*Kawina* Barton, 1916 [44].

#### Type species

*Kawina vulcanus* (Billings, 1865 [45]).

#### Diagnosis

Medial portion of S0 strongly concave anteriorly. Lateral margins of the glabella strongly converge anterior of S1. Border of the librigena narrow (tr.). Border furrow of the librigena pencil thin. For addition diagnostic criteria see the diagnosis of *K. vulcanus* in Whittington [46].

#### Discussion

Because the phylogenetic analysis indicates *Kawina* is paraphyletic, we propose that the genus be redefined as a monotypic taxon including its type species. All other species originally placed within the genus *Kawina* (or its junior synonym *Cydonocephalus*) are placed within “*Kawina*”.

*Sphaerexochus* Beyrich, 1845 [47].

#### Type species

*Sphaerexochus mirus* Beryich, 1845 [47].

#### Diagnosis

See Lane [3].

#### Discussion

Some species that have traditionally been placed within the genus *Sphaerexochus* could not be considered in phylogenetic analysis because they were poorly preserved or very incomplete. For example, *S. eximius, S. parabibrisulcatus,* and *S. trisulcatus* were excluded from the analysis because they are based only on glabellar material. *Sphaerexochus sugiyamai*, *S. planirachis*, *S. lanei, S.? shallochensis*, and *S. balclatchiensis* were excluded because their holotype specimens were strongly crushed and/or deformed. *Sphaerexochus lorum* was excluded from analysis because its pygidia and cranidia were severely crushed and eroded. *Sphaerexochus bridgei* and *S. arcuatus* were excluded from the analysis because their pygidia were incomplete and effaced. The assignment of these taxa, excluding *S.? shallochensis* (whose generic affinity cannot be determined because its type consists of a crushed thorax), to *Sphaerexochus*, however, is valid based on the presence of the following characters: wide spacing between medial tips of S1; S2 and S3 weakly incised to absent; shape of S1 straight close to the lateral glabellar margins; strongly convex anterior glabellar border; and the third axial ring of the pygidium is partially or completely fused to the terminal axial piece. In addition, *Sphaerexochus angustifrons* was treated as synonymous with *S. calvus* following Warburg [48].

The species that have been previously referred to as *S. desertus* and *S. bohemicus* were excluded from analysis because their affinities do not appear to lie with *Sphaerexochus*; in particular, the pygidium of *S. bohemicus* shows significant similarities with eccoptochilinids and *S. desertus* has been classified as an asaphid. *Sphaerexochus centeo* and *S. akimbo* unfortunately could not be considered in phylogenetic analysis.

### Biogeographic Analysis

The vicariance tree suggests a close relationship between N.W. Laurentia and Avalonia that is not replicated in the geodispersal tree. This relationship is governed by the condition of the basal node of the phylogeny, which was reconstructed as a combined E. Laurentia-N.W. Laurentia-Avalonia. The node is temporally constrained to the Early Ordovician, when Avalonia was separated from Laurentia and peripheral to Gondwana [49]. Since there is no paleomagnetic evidence to suggest that Avalonia and Laurentia were joined during the Late Cambrian/Early Ordovician, we interpret this result as being caused by a long distance dispersal event. The geodispersal tree suggests a close relationship between Avalonia and Bohemia. The pattern could potentially be the result of the movement of Bohemia towards the equatorial Laurentia-Baltica-Avalonia complex during the Late Ordovician/Silurian [49]. Previous work on the Deiphoninae, another group of cheirurid trilobites, suggests a similar geodispersal event between Laurentia and Bohemia [2]. However, based on the area cladogram, the relationship between Avalonia and Bohemia is only supported by the biogeographic states of two taxa (*S*. *britannicus* and *S*. *mirus*), so it would be prudent not to make too much of this pattern.

The geodispersal tree also suggests a close relationship between E. Laurentia and the Yangtze block. This relationship is replicated in the vicariance tree, suggesting that the processes affecting geodispersal and vicariance between these two regions were the same, potentially implicating cyclical processes such as sea-level rise and fall. However, paleomagnetic and other faunal evidence suggest that these two regions were far apart [34], [49]. The pattern may be governed by the fact that the ancestral node of the large polytomy in Figure 5 was reconstructed as a combined E. Laurentia-Yangtze, thereby resulting in each end member taxon in the polytomy being derived via vicariance or geodispersal from the combined ancestral area of E. Laurentia-Yangtze. In order to test the effects of this polytomy, the biogeographic analysis was run again but using the phylogeny that excluded *S. romingeri*. This time, the close relationship between E. Laurentia and Yangtze is only recovered in the geodispersal tree, although other area relationships within the biogeographic analysis change as well, suggesting reasonably that any relationship between E. Laurentia and Yangtze is attributable to long distance dispersal. A similar long distance dispersal event has been observed in deiphonine trilobites between N.W. Laurentia and Australia during the late Ordovician [2].

### Conclusions

A phylogenetic analysis of the sphaerexochines suggests that the genus *Sphaerexochus* is monophyletic as originally defined, while the genera *“Kawina”* and *“Cydonocephalus”* form a paraphyletic grade at the base of *Sphaerexochus*. The topology of the tree also suggests that the sphaerexochines were barely affected by the end Ordovician mass extinction event. Since the group is presumed to have had benthic larvae, this result agrees with the previous study by Chatterton and Speyer [13] on trilobite survivability across the extinction event. Compared to the extinction patterns observed in related groups, like the deiphonine trilobites [2], the sphaerexochines are particularly exceptional because they not only appear to suffer little extinction, but potentially proliferate during or immediately after the event. The biogeographic patterns of the group do not strongly suggest a biogeographic pattern in survival across the extinction event. The biogeographic analysis does suggest, however, that the sphaerexochines may have been capable of fairly long-distance dispersal, despite their benthic larval and adult life strategies. This could be the result of a planktonic-benthic larval life strategy, which has been hypothesized by Chatterton and Speyer [13] as an alternative interpretation of cheirurid ontogeny. To test this claim, further biogeographic analyses on cheirurid trilobites will be necessary to see if multiple cheirurid groups exhibit this pattern in long-distance dispersal.

## Supporting Information

Table S1 **Character state distributions for taxa used in phylogenetic analysis.** Characters and character states are as listed in the text. Missing data are indicated by “?”. Character numbers are listed at top of table.
(DOC)

Table S2 **Geodispersal matrix derived from the area cladogram in** **Figure 2** **.** “0” represents the primitive state, absent from all areas, and “1” and “2” represent the derived states, where are all characters are treated as ordered. The outgroup is the all “0” ancestor, primitively absent from all areas.
(DOC)

Table S3 **Vicariance matrix derived from the area cladogram in** **Figure 2** **.** “0” represents the primitive state, absent from all areas, and “1” and “2” represent the derived states, where are all characters are treated as ordered. The outgroup is the all “0” ancestor, primitively absent from all areas.
(DOC)
