# Supplementary material for: Phylogenetic and Biogeographic Analysis of Sphaerexochine Trilobites
Source: PLoS One. 2011 Jun 27;6(6):e21304. doi: 10.1371/journal.pone.0021304 (PMC3124496; doi:10.1371/journal.pone.0021304)
Supplement: Table S1 — Character state distributions for taxa used in phylogenetic analysis. Characters and character states are as listed in the text. Missing data are indicated by “?”. Character numbers are listed at top of table. (DOC) [file pone.0021304.s001.doc]

## Table S1

| Character/taxon | 1 | 2 | 3 | 4 | 5 | 6 | 7 | 8 | 9 | 10 | 11 | 12 | 13 | 14 | 15 | 16 | 17 | 18 | 19 | 20 | 21 | 22 | 23 | 24 | 25 | 26 | 27 | 28 | 29 | 30 | 31 | 32 | 33 | 34 | 35 | 36 | 37 | 38 | 39 |
| --- | --- | --- | --- | --- | --- | --- | --- | --- | --- | --- | --- | --- | --- | --- | --- | --- | --- | --- | --- | --- | --- | --- | --- | --- | --- | --- | --- | --- | --- | --- | --- | --- | --- | --- | --- | --- | --- | --- | --- |
| *S. latifrons* | 0 | 0 | 1 | 2 | ? | 0 | 0 | 1 | ? | 1 | 0 | 1 | 0 | 0 | 0 | 0 | ? | ? | ? | ? | 0 | 1 | 1 | 1 | 2 | 0 | 1 | 0 | 0 | 1 | 0 | 1 | 1 | 0 | 1 | 0 | 1 | 1 | 0 |
| *S. molongloensis* | 0 | 0 | 1 | 2 | ? | 0 | 0 | 1 | 0 | 1 | 0 | 1 | 0 | 0 | 0 | 0 | 1 | ? | ? | ? | 0 | 1 | 1 | 1 | 2 | ? | 1 | 0 | 0 | ? | 1 | 1 | 1 | 1 | 0 | 0 | 1 | 0 | 0 |
| *S. scabridus* | 0 | 0 | 1 | 2 | ? | 0 | 0 | 1 | ? | 1 | 0 | 1 | 0 | 0 | 0 | 0&2 | ? | 1 | 1 | ? | 0 | 1 | 1 | 1 | 1 | 0 | 0 | 0 | 0 | 1 | 0 | 1 | 1 | 0 | 1 | 0 | 1 | 1 | 0 |
| *S. atacius* | 1 | 0 | 0 | 1 | 0 | 0 | 0 | 1 | 1 | 0 | 0 | 0 | 0 | 0 | 0 | 2 | 1 | 0 | 1 | 0 | 0 | 1 | 1 | 0 | 2 | 1 | 0 | 0 | ? | ? | 0 | 1 | 0 | 1 | 0 | 1 | 0 | ? | 1 |
| *S. eurys* | 0 | 0 | 1 | ? | 0 | 0 | ? | 1 | 1 | 1 | 0 | 1 | 0 | 0 | 0 | 0 | 1 | ? | ? | 1 | 0 | 1 | 0 | 1 | 2 | 0 | 0 | 0 | 0 | ? | 1 | 1 | 0 | 1 | 1 | 1 | 1 | 0 | 1 |
| *S. calvus* | 0 | 0 | 1 | 1 | 0 | 0 | ? | 1 | 0 | 1 | 0 | 1 | 0 | 0 | 0 | 0 | 2 | ? | ? | ? | 0 | 0 | 1 | 1 | 1 | 0 | 1 | 0 | 0 | 1 | 1 | 0 | 1 | 1 | 0 | 0 | 1 | 0 | 0 |
| *S. lacianatus* | 0 | 0 | 1 | 2 | ? | 0 | 0 | 1 | 1 | 0 | 0 | 1 | 0 | 0 | 0 | 0 | 2 | 1 | 1 | 1 | 1 | 0 | 0 | 1 | 2 | 0 | 1 | 0 | 0&1 | 0 | 1 | 1 | 1 | 0 | 1 | 0 | 1 | 1 | 0 |
| *S. johnstoni* | 0 | 0 | 1 | 2 | 0 | 0 | 0 | 1 | 1 | 1 | 0 | 1 | 0 | 0 | 0 | 0 | 1 | ? | ? | ? | 1 | 1 | 0 | 1 | 2 | 0 | 1 | 0 | 0 | 0 | 1 | 0 | 1 | 1 | 1 | 0 | 1 | 0&1 | 0 |
| *S. mirus* | 0 | 0 | 1 | 2 | 0 | 0 | 0 | 1 | ? | 1 | 0 | 1 | 0 | 0 | 0 | 0 | ? | ? | ? | 1 | 1 | 0 | 1 | 1 | 2 | 0 | 1 | 0 | 0 | 1 | 1 | 0 | 1 | 1 | 1 | 0 | 1 | 1 | 0 |
| *S. britanicus* | 0 | 0 | 1 | 2 | 0 | 0 | 0 | 1 | 1 | 1 | 0 | 1 | 0 | 0 | 0 | 0 | 1 | ? | ? | ? | 0 | 1 | ? | 1 | 2 | 0 | 0 | 0 | 0 | 1 | 1 | 0 | ? | 1 | 1 | 0 | ? | 1 | 0 |
| *S. pulcher* | 0 | 0 | 1 | 1 | 0 | 0 | 0 | 1 | 0 | 0 | 0 | 1 | 0 | 0 | 0 | 0 | 1 | 0 | 1 | 0 | 1 | 1 | 1 | 1 | 2 | 1 | 0 | 1 | 1 | 1 | 0 | 1 | 1 | 1 | 1 | 0 | 1 | 0 | 1 |
| *S. parvus* | 0&1 | 0 | 1 | 1 | 0 | 0 | 0 | 1 | 0 | 0 | 0 | 1 | 0 | 0 | 0 | 0 | 1 | 1 | 0 | 0 | 0 | 1 | 1 | 0 | ? | ? | 1 | 0 | ? | ? | 0 | 1 | ? | 1 | 1 | 0 | 1 | ? | ? |
| *S. brandlyi* | 0 | 0 | 1 | 2 | 0 | 0 | 0 | 1 | 1 | 1 | 0 | ? | 0 | 0 | 0 | ? | 1 | 1 | 0 | ? | 0 | 0 | 1 | 1 | 0 | 0 | 1 | 1 | 0 | 0 | 1 | 0 | 1 | 1 | 1 | 0 | 0 | 0 | 0 |
| *S. romingeri* | 0 | 0 | 1 | 2 | 0 | 0 | 0 | 1 | 1 | 1 | 0 | 1 | 0 | 0 | 0 | 0 | 2 | ? | ? | ? | 0 | 0 | 0 | 1 | 2 | 0 | 1 | 0 | 0 | 0 | 0 | 1 | 1 | 1 | 1 | 0 | 1 | 0 | 0 |
| *S. fibrisulcatus* | 1 | 0 | ? | 2 | ? | 0 | ? | 1 | ? | 0 | 1 | 0 | 0 | 0 | 0 | ? | ? | ? | ? | ? | 0 | 1 | ? | 1 | 1 | ? | 1 | 0 | 1 | ? | 1 | 1 | 1 | 1 | 0 | 0 | ? | 0 | 1 |
| *S. hapsidotus* | 0&1 | 0 | 1 | 1 | 0 | 0 | 0 | 1 | 0&1 | 0 | 0 | 1 | 0 | 0 | 0 | 0 | 1 | 1 | 1 | 0 | 1 | 0 | 0 | 1 | 2 | 1 | 0 | 0 | 1 | 0 | 0 | 1 | 1 | 1 | 0 | 1 | 0 | 0 | 0 |
| *S. glaber* | 0 | 0 | 1 | 2 | ? | 0 | ? | 1 | ? | 1 | 0 | 1 | 0 | 0 | 0 | 0 | ? | ? | ? | ? | 0 | 0 | 0 | 1 | 1 | ? | 0 | 0 | 0 | 0 | 0 | 0 | 1 | 1 | 1 | 0 | 1 | 0 | 0 |
| *S. arenosis* | 0&1 | 0 | 1 | 1 | 0 | 0 | 0 | 1 | 1 | 0 | 0&1 | 0 | 0 | 0 | 0 | 2 | 2 | 1 | 0 | 0 | 1 | 0 | 0 | 1 | 2 | ? | 0 | 1 | 1 | ? | 1 | 1 | 0 | 1 | 1 | 1 | 0 | 0 | 1 |
| *S. dimorphus* | 0 | 0 | 1 | 2 | 0 | 0 | 0 | 1 | 1 | 1 | 0 | 1 | 0 | 0 | 0 | 0 | 1 | 1 | 1 | 1 | 0&1 | 1 | 0 | 1 | 2 | 0 | 0&2 | 0 | 0 | 0&1 | 0 | 1 | 1 | 0 | 1 | 0 | 1 | 1 | 0 |
| *S. hiratai* | 0 | 0 | 1 | 2 | ? | 0 | ? | 1 | ? | 1 | 0 | 1 | 0 | 0 | 0 | 0 | ? | ? | ? | ? | 0 | 1 | 1 | 1 | 2 | 0 | 1 | 1 | 0 | 1 | 1 | 1 | 1 | 0 | 0&1 | 0 | 1 | 1 | 0 |
| *K. vulcanus* | 1 | 1 | 0 | 0 | 1 | 1 | 1 | 0 | 1 | 0 | 0 | 0 | 0 | 0 | 1 | 1 | 0 | ? | ? | ? | 0 | 1 | 1 | 0 | 2 | 1 | 2 | 0 | 1 | ? | 1 | 1 | 0 | 1 | 1 | 1 | 1 | 0 | 2 |
| *“K.” arnoldi* | 1 | 1 | 0 | 0 | 1 | 1 | 1 | 0 | 0 | 0 | 0 | 0 | 0 | 0 | 0 | 1 | 0 | 1 | 1 | 1 | 0 | 1 | 1 | 0 | 2 | 1 | 2 | 0 | 1 | 1 | 1 | 1 | 1 | 1 | 1 | 0 | 1 | 0 | 2 |
| *“K.” divergens* | 0&1 | 1 | 0 | 0 | ? | 1 | 1 | 0 | ? | 0 | 0 | 0 | 0 | 0 | 1 | 0 | ? | ? | ? | ? | 1 | 1 | 1 | 0 | 2 | 1 | 0 | 1 | 1 | ? | 1 | 1 | 0 | 1 | 1 | 1 | 1 | 0 | 2 |
| *“K.” torulus* | 1 | 1 | 1 | 0 | 1 | 0 | 1 | 0 | 1 | 0 | 0 | 0 | 0 | 0 | 0 | 1 | 1 | 1 | 1 | 1 | ? | ? | ? | ? | ? | ? | ? | ? | ? | ? | ? | ? | ? | ? | ? | ? | ? | ? | ? |
| *“K.” griphus* | 1 | 1 | 1 | 0 | 1 | 0 | 0 | 0 | 1 | 0 | 0 | 0 | 0 | 0 | 0 | 1 | 1 | 1 | 1 | 1 | ? | ? | ? | ? | ? | ? | ? | ? | ? | ? | ? | ? | ? | ? | ? | ? | ? | ? | ? |
| *“K.” prolificus* | 1 | 1 | 1 | 0 | 1 | 0 | 0 | 0 | ? | 0 | 0 | 0 | 0 | 0 | 0 | 0 | ? | ? | ? | ? | ? | ? | ? | ? | ? | ? | ? | ? | ? | ? | ? | ? | ? | ? | ? | ? | ? | ? | ? |
| *“K.” scrobiculus* | 1 | 1 | 1 | 1 | ? | 0 | 0 | 0 | 1 | 0 | 0 | 1 | 0 | 1 | 0 | 0 | 2 | ? | ? | ? | ? | ? | ? | ? | ? | ? | ? | ? | ? | ? | ? | ? | ? | ? | ? | ? | ? | ? | ? |
| *“K.” mercurius* | 1 | 1 | 1 | 0 | ? | 1 | 1 | 0 | 1 | 0 | 0 | 0 | 1 | 1 | 0 | 0 | 0 | ? | ? | ? | ? | ? | ? | ? | ? | ? | ? | ? | ? | ? | ? | ? | ? | ? | ? | ? | ? | ? | ? |
| *“K.” prominulus* | 1 | 1 | 1 | 0 | ? | 1 | 1 | 0 | ? | 0 | 0 | 0 | 1 | 1 | 0 | 0 | ? | ? | ? | ? | ? | ? | ? | ? | ? | ? | ? | ? | ? | ? | ? | ? | ? | ? | ? | ? | ? | ? | ? |
| *“K.” limbata* | 1 | 1 | 0 | 0 | 1 | 1 | 1 | 0 | 0 | 0 | 0 | 0 | 0 | 0 | 1 | 1 | 0 | ? | ? | ? | ? | ? | ? | ? | ? | ? | ? | ? | ? | ? | ? | ? | ? | ? | ? | ? | ? | ? | ? |
| *“K.” sexapugia* | 1 | 1 | 0 | 0 | 0 | 1 | 0 | 0 | 0 | 0 | 0 | 0 | 0 | 0 | 0 | 0 | 0 | 1 | 0 | 1 | 1 | 1 | 1 | 2 | 2 | 1 | 2 | 0 | 1 | 1 | 1 | 1 | 0 | 1 | 1 | 1 | 1 | 0 | 2 |
